# Calcitonin gene-related peptide antagonists versus botulinum toxin A for the preventive treatment of chronic migraine protocol of a systematic review and network meta-analysis: A protocol for systematic review

**DOI:** 10.1097/MD.0000000000018929

**Published:** 2020-01-31

**Authors:** Tianwei She, Yaoyao Chen, Taichun Tang, Min Chen, Hui Zheng

**Affiliations:** a The Third Hospital/Acupuncture and Tuina School, Chengdu University of Traditional Chinese Medicine; b Department of Anorectal Diseases, Hospital of Chengdu University of Traditional Chinese Medicine, Chengdu, China.

**Keywords:** botulinum toxin A, calcitonin gene-related peptide antagonists, chronic migraine, network meta-analysis, study protocol

## Abstract

**Background:**

Although calcitonin gene-related peptide antagonists and botulinum toxin A have been shown efficacy in preventing chronic migraine, there is no direct evidence for their comparative effectiveness. This review is to assess the comparative effectiveness and safety of calcitonin gene-related peptide antagonists and botulinum toxin A for chronic migraine using network meta-analysis.

**Methods:**

OVID MEDLINE, EMBASE, and Cochrane Central Register of Controlled Trials will be searched for relevant randomized controlled trials from their inception to December 2019 without language restriction. We will include trials testing the effectiveness of calcitonin gene-related peptide antagonists or botulinum toxin A in patients with chronic migraine. The outcomes are mean change from baseline in the number of headache days, the mean change from baseline in the number of migraine days, the mean change from baseline in headache hours, responder rate, and adverse events rate. The methodological quality of the included randomized controlled trials will be evaluated using Cochrane Collaboration's risk of bias tool. Standardized mean difference will be used to synthesize continuous variables and risk ratio will be used to synthesize categorical variables. Pairwise and network meta-analysis will be performed using a frequentist method in netmeta package (R 3.5.0, www.r-project.org).

**Results:**

Ethical approval and informed consent are not required for this systematic review. The results will be submitted to a peer-reviewed journal and conference abstracts for publication.

**Conclusion:**

The result of the review will systematically provide suggestions for clinicians, patients, and policy makers in the treatment of chronic migraine.

PROSPERO registration number: CRD42018089201.

## 1 Introduction

Migraine is a neurological disorder characterized by periodic attacks of headache and often accompanied by a series of reversible systemic symptoms such as photophobia, phonophobia, vertigo, nausea, and vomiting.^[1]^ The occurrence of headache on more than 15 days per month, and that at least 8 days meet diagnostic criteria of migraine is defined as chronic migraine.^[2]^ The population prevalence of chronic migraine ranged from 0.9% to 5.1%,^[3]^ of which 3/4 is female.^[4]^ Patients with chronic migraine often reported substantially impaired quality of life, decreased productivity, and rising costs of health-care.^[5–7]^ Because of huge social and economic burden, chronic migraine ranks one of the most prevalent and disabling medical illnesses in the world.^[8]^

The primary goal of chronic migraine treatment is to reduce the impact of disease on patient's lives. So, it is necessary to decrease the frequency and duration of headache attacks and reduce migraine-related disability.^[2,9]^ Traditional preventive medications, for example, antidepressants, β blockers, calcium-channel blockers, and anticonvulsants can reduce the frequency of attacks by about 50%; however, accompanied with intolerable side effects.^[10]^

Guidelines recommended botulinum toxin A (BoNT-A) as an effective and well-tolerated treatment prophylactic medication for chronic migraine.^[11,12]^ The mechanisms of BoNT-A for chronic migraine may include modulation of neurotransmitter release, changes in surface expression of receptors and cytokines as well as enhancement of opioidergic transmission.^[13]^ Peripheral injection 155 U195 U to 31–39 sites every 12-weeks has proved to be effective.^[12]^

Recent studies have shown that calcitonin gene-related peptide (CGRP) is a key factor in the pathogenesis of migraine.^[14]^ CGRP antagonists can significantly improve the treatment effect of chronic migraine,^[15]^ this may expected to attract 20% of migraine patients who do not respond to existing treatments.^[16]^ Several pivotal trials have proved that anti-CGRP monoclonal antibodies to be well tolerated and efficacious for the prevention of chronic migraine. And erenumab-aoo, an anti-CGRP monoclonal antibodies, has also been approved by the US Food and Drug Administration as a preventive treatment for chronic migraine.^[17–20]^

Heretofore, there is no trial to directly compare BoNT-A with CGRP antagonists, so in order to provide clinicians and patients with the best treatment decisions, we will conduct a systematic review and network meta-analysis to indirectly compare the effectiveness of 2 medications and answer the following questions: Which is better and safer in reducing the attacks of headache?

## 2 Methods

### 2.1 Study design and registration

The systematic review and network meta-analysis will assess the comparative effectiveness and safety of CGRP antagonists and BoNT-A for migraine prophylaxis. We will conduct this network meta-analysis in accordance with preferred reporting items for systematic review and meta-analysis protocols.^[21]^ The network meta-analysis has been registered in PROSPERO (https://www.crd.york.ac.uk/PROSPERO/, CRD), PROSPERO registration number is CRD42018089201.

### 2.2 Information sources

We will search OVID MEDLINE, EMBASE, the Cochrane Central Register of Clinical Trials from inception to December 2019 without language restriction, which examine the effectiveness of the CGRP antagonists and BOTOX interventions for chronic migraine prophylaxis. To find out randomized controlled trials (RCTs) that examined the effectiveness of the CGRP antagonists and BoNT-A for chronic migraine prophylaxis, we will develop a search strategy by using a combination of terms of medical subject headings (MeSH) and keywords. MeSH and keywords contain “chronic migraine,” “randomized controlled trials,” and synonymous words. Details of the search strategy are provided in Table 1. Systematic reviews examining the effect of CGRP antagonists and BoNT-A on chronic migraine will be retrieved, the reference of which will be screened for relevant RCTs.

**Table 1:**
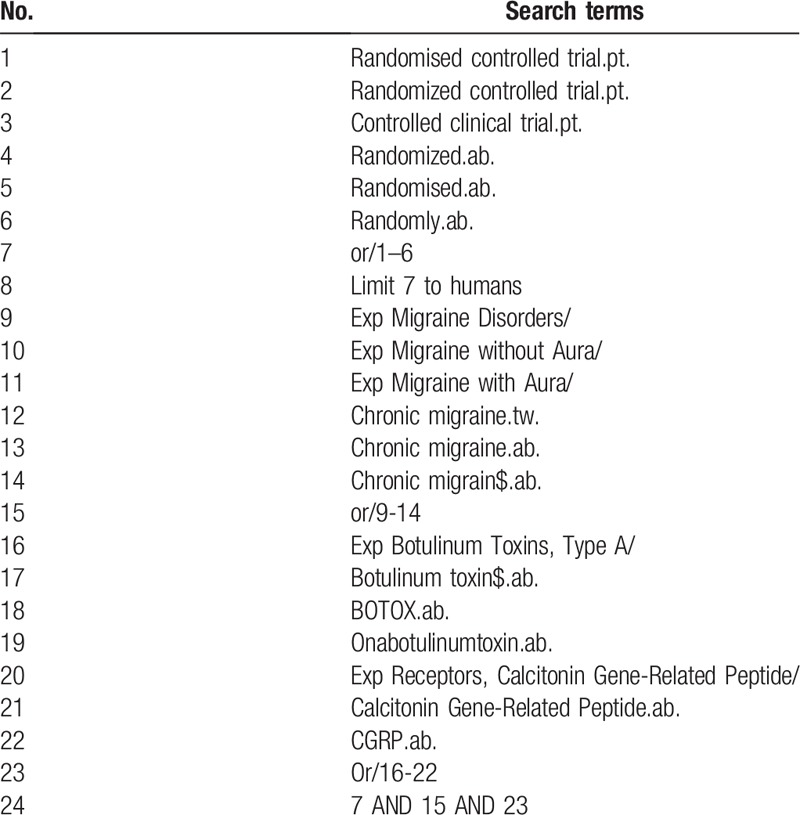
Search strategy.

### 2.3 Inclusion and exclusion criteria

#### 2.3.1 Studies design

We will include trials with randomized controlled design and exclude specific design of randomized controlled trials like the N-of-1 design and the cross-over design. Besides, the cohort studies, case reports, case series and experimental studies that focus on treatment mechanism will be excluded.

#### 2.3.2 Participants

We will include males or females who meet the following conditions: with chronic migraine diagnosed according to the International Classification of Headache Disorders (ICHD 2nd edition or ICHD 3rd edition); with headache attacks for at least 15 days per month and migraine attacks for at least 8 days per month. Patients with suspected headaches due to trauma, elevated blood pressure, or other organic diseases will be excluded.

#### 2.3.3 Interventions

We will include trials testing the effectiveness of CGRP antagonists or BoNT-A. To ensure the comparability between CGRP antagonists and BoNT-A, we will include trials using injections only. Moreover, we will include trials comparing CGRP antagonists with placebo and trials comparing BoNT-A with placebo. Trials comparing CGRP antagonists or BoNT-A with positive drug control will be excluded.

#### 2.3.4 Outcome measures

The primary outcome was the mean change from baseline in the number of headache days during a 4-week assessment period (week 9–12). Secondary outcomes include the mean change from baseline in the number of migraine days, the mean change from baseline in headache-hours, the responder rate, and the rate of adverse events at week 4, 8, and 12. According to the IHS guideline,^[22]^ if the headache lasts more than 4 hours a day, it is defined as a migraine day. The responder rates have been defined as a ≥50% reduction from baseline in number of migraine days.

### 2.4 Study selection and data extraction

The process of study selection and data extraction will be performed and cross-checked by 2 independent reviewers. First, studies will be selected by reviewing the titles and abstracts according to the included criteria mentioned above. Any discrepancy in this procedure will be settled by team discussion or being arbitrated by a third reviewer. Then, possible candidates will be searched and downloaded for full-text copies for further evaluation and to determine the final included studies. At last, necessary information will be extracted from the included RCTs using a standard form, which is developed by a consensus of all the reviewers. The form covers the following domains: study ID, settings, baseline characteristics of each trial (sample size, age, sex ratio), number of study centers and groups, allocation ratio, the name of intervention and control, treatment duration, treatment frequency, and outcome assessments (primary outcome and secondary outcomes, measurement time point, the rate of adverse events). For trials with the missing data, we will contact the original authors for more information about data by email or phone calls. After all the data is extracted from the included RCTs, a third reviewer will check the completeness and correctness of the data, to ensure an accurate result of this study.

### 2.5 Risk of bias assessment

Cochrane Collaboration's risk of bias tool^[23]^ will be used to evaluate the methodological quality of the included RCTs. The risk of bias tool focuses on 6 domains: sequence generation, allocation concealment, blinding, incomplete data, selective reporting, and other bias. Two independent investigators will use the risk of bias tool to independently evaluate the quality of RCTs. Disagreements in this procedure will be settled by discussion or be judged by a third reviewer.

### 2.6 Data synthesis

We will qualitatively summarize included trials, describing direct and indirect comparisons, listing the trial design and characteristics. Before performing meta-analysis, we will assess whether the heterogeneity is significant between trials by using a cut-off point of *I*^2^ = 50%. Missing values that cannot be acquired from the authors will be calculated through the available coefficients in reference to the Cochrane handbook.^[24]^ The potential impact of these missing data on the results of the network meta-analysis will be tested in sensitivity analysis.

We will perform conventional pairwise comparisons and calculate the effect sizes and related 95% confidence intervals. We will calculate the effect sizes of continuous data with standardized mean difference (SMD), and categorical data with risk ratio. SMDs are recognized as small, median, and large effect size by using 0.2, 0.5, and 0.8 as cut-off points, respectively.^[25]^ RCTs containing a treatment with zero event will be excluded from the meta-analysis.

We will perform a network meta-analysis to compare CGRP antagonists with BoNT-A by using a frequentist method in netmeta package (R 3.5.0, www.r-project.org). Consistency of the network will be assessed by using the Cochran *Q* test, and the source of inconsistency will be investigated by a design-by-treatment decomposition method.

Subgroup analysis will be performed to each individual of CGRP antagonists. We will perform meta-regression to determine source of heterogeneity like age, duration of migraine headaches, medication overuse, and types of migraine (with or without aura).

We will perform sensitivity analysis to the factors leading to significant heterogeneity. We will exclude studies with high or unclear risk of bias to check if the results were consistent with the primary analysis; we will also exclude studies with small sample size (n < 100 per group) to check if the results were consistent.

## 3 Discussion

We hope that we can integrate direct and indirect evidence about the effectiveness of CGRP antagonists and BoNT-A for chronic migraine prophylaxis and provide a ranking by using network meta-analysis. We expect that the results will help the physicians and chronic migraine patients to choose their most appropriate and best method according to their preferences and conditions. Of course, we also hope that the results will be of interest and adoption to the policy makers, so as to the best method would be covered by health insurance.

## Author contributions

**Conceptualization:** Tianwei She, Hui Zheng.

**Data curation:** Min Chen, Hui Zheng.

**Formal analysis:** Min Chen, Hui Zheng.

**Investigation:** Yaoyao Chen, Taichun Tang

**Methodology:** Tianwei She, Hui Zheng.

**Writing – original draft:** Tianwei She, Yaoyao Chen, Min Chen, Hui Zheng.

**Writing – review & editing:** Tianwei She, Yaoyao Chen, Min Chen, Hui Zheng.

Hui Zheng orcid: 0000-0002-0494-1217.
